# A methodology for assessing the effect of correlations among muscle synergy activations on task-discriminating information

**DOI:** 10.3389/fncom.2013.00054

**Published:** 2013-05-13

**Authors:** Ioannis Delis, Bastien Berret, Thierry Pozzo, Stefano Panzeri

**Affiliations:** ^1^Robotics, Brain and Cognitive Sciences Department, Istituto Italiano di TecnologiaGenoa, Italy; ^2^Communication, Computer and System Sciences Department, Doctoral School on Life and Humanoid Technologies, University of GenoaGenoa, Italy; ^3^Institute of Neuroscience and Psychology, University of GlasgowGlasgow, UK; ^4^UR CIAMS, EA 4532 – Motor Control and Perception Team, Université Paris-Sud 11Orsay, France; ^5^Institut Universitaire de France, Université de Bourgogne, Campus UniversitaireUFR STAPS Dijon, France; ^6^INSERM, U1093, Action Cognition et Plasticité SensorimotriceDijon, France; ^7^Center for Neuroscience and Cognitive Systems, Istituto Italiano di TecnologiaRovereto, Italy

**Keywords:** muscle synergies, correlations, information theory, task decoding, single-trial analysis

## Abstract

Muscle synergies have been hypothesized to be the building blocks used by the central nervous system to generate movement. According to this hypothesis, the accomplishment of various motor tasks relies on the ability of the motor system to recruit a small set of synergies on a single-trial basis and combine them in a task-dependent manner. It is conceivable that this requires a fine tuning of the trial-to-trial relationships between the synergy activations. Here we develop an analytical methodology to address the nature and functional role of trial-to-trial correlations between synergy activations, which is designed to help to better understand how these correlations may contribute to generating appropriate motor behavior. The algorithm we propose first divides correlations between muscle synergies into types (noise correlations, quantifying the trial-to-trial covariations of synergy activations at fixed task, and signal correlations, quantifying the similarity of task tuning of the trial-averaged activation coefficients of different synergies), and then uses single-trial methods (task-decoding and information theory) to quantify their overall effect on the task-discriminating information carried by muscle synergy activations. We apply the method to both synchronous and time-varying synergies and exemplify it on electromyographic data recorded during performance of reaching movements in different directions. Our method reveals the robust presence of information-enhancing patterns of signal and noise correlations among pairs of synchronous synergies, and shows that they enhance by 9–15% (depending on the set of tasks) the task-discriminating information provided by the synergy decompositions. We suggest that the proposed methodology could be useful for assessing whether single-trial activations of one synergy depend on activations of other synergies and quantifying the effect of such dependences on the task-to-task differences in muscle activation patterns.

## Introduction

The central nervous system (CNS) is capable of performing a wide repertoire of motor tasks despite the high complexity of the musculoskeletal system (Bizzi et al., 1998). A possible strategy for achieving this accuracy despite the difficulties of controlling so many degrees of freedom may rely on generating movement as combination of a small number of invariant muscle patterns, commonly referred to as muscle synergies (Tresch et al., 1999; D'Avella et al., 2003; Bizzi et al., 2008). Muscle synergies are presumably recruited by neural motor commands—the so-called synergy activations—to produce the muscle activities required for task execution (Ting and McKay, 2007). However, how the appropriate synergies are combined and their corresponding activation levels are selected in single trials is still an open question.

An important empirical observation is that, although the CNS is able to generate a reliable and consistent motor behavior in each single trial, synergy activations are highly variable across trials: repeated executions of the same motor task rely on different activations of muscle synergies (Tresch and Jarc, 2009). The impact of such trial-to-trial variability on task performance remains to be understood. A question of particular interest is whether activations of different synergies in the same trials are correlated—in other words, whether recruitment of a given synergy may depend not only on the task at hand but also on the activation of other synergies—and what the potential roles of such correlations among synergy activations may be (Saltiel et al., 2001; Tresch et al., 2006). These trial-to-trial correlations of synergy activations are often neglected in studies that report for example only the mean of each synergy activation coefficient independently of the other. Investigating such correlations requires, of course, ways to quantify their joint distributions across different trials.

From the theoretical point of view, these correlations may arise from different factors or serve a number of different purposes. Because individual synergies are presumably recruited by different neural drives that are not necessarily synchronous, it is conceivable that information about the recruitment of one synergy may be utilized for the activation of complementary synergies. In the same vein, the brain could rely on such correlations to cope with neural noise by reinforcing the relationships between the recruitment of the different synergies. Moreover, the correlation between synergy activations could be an emergent constraint of the task execution and its identity. For example, variations of speed in a set of arm pointing movements to a given spatial target could naturally lead to such correlations. Alternatively, these correlations may not serve a purposeful function, but rather arise from limitations of the neural-musculoskeletal systems and be detrimental to task performance. In such latter case, a useful strategy would be to minimize such correlations rather than using them as part of the motor strategy.

Here we introduce an analytical methodology to address the nature and functional role of trial-to-trial correlations between synergy activations. This method, which takes inspiration from methodologies derived for studying neural population codes, is designed to quantify how these correlations may contribute to generating appropriate motor behavior in single trials. The algorithm we propose first divides correlations between muscle synergies into types (noise correlations, quantifying the trial-to-trial covariations of synergy activations at fixed task, and signal correlations, quantifying the similarity of task tuning of the trial-averaged activation coefficients of different synergies). Then, building on recent work designed to quantify single-trial task discriminability of EMG data (Delis et al., 2013) the method employs single-trial methods (task-decoding and information theory) to quantify the overall effect of correlations between synergy activations on the task-to-task differences in patterns of muscle activation. We show that the methodology is readily applicable to any type of synergy decomposition and demonstrate its use for addressing the functional role of coordinated synergy recruitment on a task-by-task basis. To illustrate the method, and to begin to reason about the existence and potential function of cross-synergy correlations in real EMG data, we finally implement the method on muscle synergies extracted from an electromyographic (EMG) dataset recorded during the execution of a variety of reaching tasks (Delis et al., 2013). This application reveals the robust presence of information-enhancing patterns of signal and noise correlations among pairs of synchronous synergies, and shows that they contribute to enhance by approximately 9–15% (depending on the set of tasks considered) the task-discriminating information provided by synchronous synergy decompositions. Activations of time-varying synergies were instead much more weakly correlated and their correlations had a more limited impact on task information (0–5%).

## Materials and methods

### Muscle synergy extraction

The extraction of muscle synergies relies on dimensionality reduction algorithms that determine stereotyped muscle activation patterns from the EMG data by modeling muscle activities as linear combinations of the extracted synergies (D'Avella et al., 2003; Tresch et al., 2006; Tresch and Jarc, 2009). There exist two influential models for describing muscle patterns as synergy combinations: the time-varying synergies (D'Avella and Tresch, 2002), which are genuine spatiotemporal patterns of muscle activation, with the EMG output specified by the amplitude and time lag of the recruitment of each synergy; and the synchronous synergies, which are co-varying groups of muscle activations, with the EMG output specified by a temporal profile defining the timing of each synergy during the task execution (Tresch et al., 1999; Cheung et al., 2005). Here, we implemented both models using algorithms based on Non-negative Matrix Factorization (NMF).

We selected this dimensionality reduction technique over alternatives, such as Principal Component Analysis (PCA) or Independent Component Analysis (ICA), because of two reasons. First, it imposes a non-negativity constraint to the extracted synergies (Tresch et al., 2006). Such a constraint reflects well the properties of muscle activation signals, as muscles cannot be activated “negatively.” Second and more importantly for this study, PCA and ICA make specific assumptions about the dependencies among the extracted synergies (orthogonality and statistical independence respectively), which impose constraints on the relationships between the corresponding synergy activations as well. NMF does not impose such constraints and thus seems more suitable for studying the trial-to-trial relationships of synergy activations.

#### Synchronous synergy model

We used the NMF algorithm (Lee and Seung, 1999) to extract synchronous synergies. In this model, the EMGs are represented as a linear combination of a set of time-invariant activation balance profiles across all muscles activated by a time-dependent activation coefficient:

(1) $$m^{s}(t)=\sum_{i\;=\;1}^{N}c_{i}^{s}(t)w_{i}+\varepsilon^{s}(t)$$

where ***m***^*s*^(*t*) is again the EMG data of all muscles at time *t*; ***w***_*i*_ is the synergy vector for the *i*-th synergy; *c*^*s*^_*i*_(*t*) is the scalar coefficient for the *i*-th synergy at time *t*; *N* is the total number of synergies composing the dataset; and **ε**^*s*^(τ) is the residual (e.g., noise). The exponent *s* indicates the trial-dependence of the variables.

#### Time-varying synergy model

We used the time-varying synergy model first introduced in D'Avella and Tresch (2002). According to it, a muscle pattern recorded during one sample *s* is decomposed into *N* time-varying muscle synergies combined as follows:

(2) $$m^{s}(t)=\sum_{i\;=\;1}^{N}c_{i}^{s}w_{i}\left(t-t_{i}^{s}\right)+\varepsilon^{s}(t)$$

where ***m***^*s*^(*t*) is a vector of real numbers, each component of which represents the activation of a specific muscle at time *t*; ***w***(τ) is a vector representing the muscle activations for the *i*-th synergy at time τ after the synergy onset; *t*^*s*^_*i*_ is the time of synergy onset; *c*^*s*^_*i*_ is a non-negative scaling coefficient; and **ε**^*s*^(τ) is the residual (e.g., noise). To implement this model, we used the NMF-based time-varying synergy extraction introduced in D'Avella et al. (2003).

In the following, our purpose is to develop a mathematical procedure for quantifying the single-trial correlations among synergy activations and in particular their contribution to the task-discriminating information carried by the synergy decompositions.

### Single-trial decoding of motor tasks in the muscle synergy space

In our previous work, we introduced an approach for predicting the motor task performed in every single-trial using muscle synergy activation parameters (Delis et al., 2013). In this study, our aim is to examine the functional role of the correlations between muscle synergy activations in each single trial. For simplicity, we restricted our decoding analysis to one single-trial parameter per synergy. We use as decoding parameters the time-integral of the synergy activation coefficient for the synchronous synergies and the scaling coefficient for the time-varying synergies. Decoding was performed using a quadratic discriminant algorithm (QDA) (Duda et al., 2001). QDA assumes that the probability of obtaining the synergy activation vector given that task *t* is performed follows a Gaussian distribution for each task. Based on this assumption, the algorithm attempts to decode the task by determining the decision boundaries that maximize the ratio of the between-task over the within-task distances. In contrast to the linear discriminant algorithm that we used previously, QDA assumes unequal covariance matrices across tasks and this difference leads to quadratic instead of linear decision boundaries (Duda et al., 2001). Although the differences between these two algorithms in terms of task decoding were relatively small, the use of QDA can in principle better handle the differences between correlated and uncorrelated data, as strong correlations may lead to curved decision boundaries (Duda et al., 2001; Averbeck and Lee, 2006).

To validate decoding results, we implemented the “leave-one-out” cross-validation (Kjaer et al., 1994; Quian Quiroga and Panzeri, 2009), in which each trial is predicted based on the distribution of all other trials. Hence, in each step of this cross-validation procedure the training set consists of *M* − 1 trials, while the test set consists of 1 trial. This process is repeated until all *M* trials have been tested. The “leave-one-out” approach maximizes the number of trials used for optimizing the decoder (training set) as well as for assessing its performance (test set).

### Quantifying information from the confusion matrix

Once decoding is performed, we need to choose a measure of decoding performance. The simplest possibility, which we used in our earlier work, is to use the percentage of correct decoding (Delis et al., 2013). A potential problem with using percent correct is that it may fail to capture all task-discriminating information even when using an optimal decoding algorithm. Synergy coefficients may convey information by means other than just reporting the most likely task given the muscle synergy activation pattern: for example, they can provide the information that some tasks are utterly unlikely based on the synergy activations (Quian Quiroga and Panzeri, 2009).

A way to include in our calculation more ways to encode information is to use the mutual information *I*(*T*;*T*^*P*^) between the actual and the predicted tasks from the decoding outcomes (Shannon, 1948). The information *I*(*T*;*T*^*P*^) is a measure of the overall information about which task out of a set is gained by the prediction of the most likely task from the single-trial muscle synergy activations, and is defined as the mutual information between the rows and columns of the so called “confusion matrix” (i.e., the matrix quantifying the probability of predicting a given task given the presentation of a certain task):

(3) $$I\left(T;T^{P}\right)=\sum_{t,t^{p}}P\left(t,t^{P}\right)\log_{2}\frac{P\left(t,t^{P}\right)}{P(t)P\left(t^{P}\right)}$$

where *t* is the motor task performed, *t*^*p*^ is the one predicted by our decoding algorithm, *P*(*t*) is the probability of execution of task *t*, *P*(*t*^*p*^, *t*) is the confusion matrix, i.e., the joint probability of predicting task *t*^*p*^ and executing task *t*, and *P*(*t*^*p*^) is the probability of predicting task *t*^*p*^ across all tasks in the considered set. Such measure of information *I*(*T*;*T*^*P*^) is not an absolute property of the synergy set, but it depends on the specific set of tasks considered (because it measures discriminability between tasks belonging to the specific set considered). In this article we will compute information about three possible task sets: the set of all eight different reaching tasks; the set of all four center-out tasks, and the set of all four out-center tasks. All quantities are computed from Equation (3), but summing over the appropriate set or subset of tasks.

It is useful to remind that information is measured in bits. Every bit of information reduces the overall uncertainty about the task by a factor of two. Perfect knowledge about the task from the synergy decomposition gives maximum mutual information of log_2_ *K*, where *K* is the number of tasks. For a given percent correct value, more information can be obtained if incorrect predictions are concentrated into clusters around the correct stimulus rather than distributed randomly (Panzeri et al., 1999b; Samengo, 2002). This information measure can reveal the effect of such systematic errors in the decoder and thus capture also this form of information that may be carried by muscle synergy activations. Information values were computed using the Information Breakdown Toolbox (Magri et al., 2009) available at www.infotoolbox.org. To eliminate the systematic bias from which information measures suffer when computed from small datasets, we used the Panzeri-Treves (PT) bias-correction method (Panzeri and Treves, 1996; Panzeri et al., 2007).

In Results, for simplicity we will abbreviate *I*(*T*; *T*^*P*^) simply as *I*.

### Selecting the set of synergies that carry all task-discriminating information in the muscle synergy space

**Figure 1:**
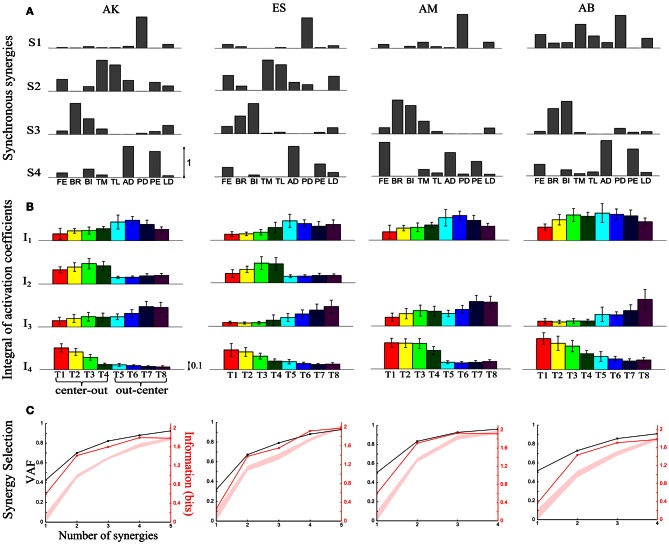
**Application of our information-based method to the synchronous synergies extracted from the EMG data of all four subjects recorded during the execution of an arm pointing task. (A)** The sets of synchronous synergies obtained from the experimental data of four subjects as vectors of activation levels of nine shoulder and elbow muscles for each subject. **(B)** Histograms of the integral of the activation coefficient (*I*) across the eight motor tasks performed. **(C)** VAF (black curve whose scale is indicated in left *y*-axis) and information curves (red curve whose scale is indicated in right *y*-axis). The shaded area represents the 5–95% confidence interval of the bootstrap test for decoding (see Materials and Methods).

The number of synergies (*N*) used to perform a set of motor tasks and their contribution to task-discriminating variations, is unknown a priori. To address this, we developed an automated procedure to select the minimal number of synergies that carry all information about task-to-task differences. This model selection technique is based upon the progressive evaluation of the statistical significance of the task-discriminating information added when progressively increasing the number of synergies in the decomposition model. After evaluating the information carried by *N* = 1 synergy, the number of synergies in the decomposition model increases step by step, until the increase of synergies does not gain any further statistically significant information. The test of statistical significance was designed as follows. For a given value *N*, we compare the information carried by the synergy parameters when using the *N* synergies with the decoding performance of the parameters of all subsets consisting of *N* − 1 synergies plus the parameters of the *N*-th synergy pseudo-randomly permuted (“shuffled”) across conditions. We repeat this shuffling procedure a number of times (100 in our implementation) to obtain a non-parametric distribution of decoding performance values in the null hypothesis that the additional synergy does not add to information carried by the synergy decomposition. In the following we evaluated this significance at the *p* < 0.05 threshold. The statistical threshold for significant increase of decoding performance was graphically highlighted in the information curves as a function of the number of synergies as a shaded area indicating the 95% confidence intervals constructed using this bootstrap procedure (Figure 1C). The selected number of synergies can be simply visualized as the smallest value of *N* for which information lies above the no-significance (shaded) area. In this way, the chosen set of *N* synergies is the smallest decomposition that captures all available task-discriminating information within the synergy space.

We verified, by means of generation of realistically simulated EMG datasets [based on linear combination of realistic synchronous and time-varying synergies with the addition of various kinds of physiologically-relevant noise—see (Delis et al., 2013)] that the method needed only a small number of trials per task (10 or more) to individuate correctly the set of synergies generating the data and to evaluate correctly their information content [data not shown, but see (Delis et al., 2013) for similar evaluations of robustness of decoding algorithms].

### Defining and quantifying signal and noise correlations among synergy activations

Before we present our methodology based on the metrics defined so far, we explain what we mean by correlations among activations of muscle synergies, how we can quantify these correlations and how they may impact on the information about the task. In general, we would like to distinguish between different kinds of correlations (“signal” and “noise”—see below) that, as shown in previous studies of neural population codes and reviewed below, are known to have different impacts on information about the task (Averbeck and Lee, 2006; Averbeck et al., 2006; Ince et al., 2010).

To separate out the contribution of task modulation and of variability not attributable to task-to-task differences, it is useful to characterize the activation of each synergy in each trial as “signal plus noise” (Gawne and Richmond, 1993; Panzeri et al., 1999a; Averbeck et al., 2006), where we refer as the “signal” the trial-averaged synergy activation coefficients for each task and took as the “noise” the trial-by-trial fluctuations of the activation response around their averaged across trials at fixed task. We stress that such “noise” does not necessarily reflect only noise in the real sense, but comprises all types of variations at fixed task, which may well include the effect of various types of potentially important contributions such as modulations arising from variations across trials of the movement kinematics.

We performed a linear analysis of correlations across synergies of both the signal and the noise, as follows. The correlation of the averaged (across all trials to the same task) synergy activation coefficients across different tasks of two given synergies are called “signal correlations” (Gawne and Richmond, 1993; Panzeri et al., 1999a; Averbeck et al., 2006) because they are entirely attributable to task-to-task variations. The signal correlation coefficient was computed, for each synergy pair and channel, as the Pearson correlation across stimuli of the trial-averaged responses. Positive values indicate that the two synergies have similar task preference, whereas a zero value indicates that the two synergies have a radically different task tuning.

Correlations manifested as covariations of the trial-by-trial fluctuation around the mean response to the task are called “noise correlations” (Gawne and Richmond, 1993; Panzeri et al., 1999a; Averbeck et al., 2006). Since these noise covariations are measured at fixed task, they ignore all effects attributable to common task-to-task variations. To quantify the strength of noise correlations, we computed the Pearson correlation coefficient (across trials at fixed task) of the trial-average-subtracted synergy coefficients. This quantifies the correlations of the variations around the mean at each trial and task. Positive values of noise correlation mean that when the activation of one synergy fluctuates over its mean values, the activation of the other synergy is also more likely to do so.

The division in signal and noise correlation is important because, as we shall see below, they have a different impact on information about tasks.

### Measuring how correlations between synergy activations affect task-discriminating information

After having defined correlations, we proceed to present a methodology for characterizing how they affect the total task information carried by the set of synergies. In other words, we aim at comparing the information available in the set of synergies including correlations between them [denoted*I*, and defined in Equation (3)] with the information that would be available if correlations were absent (Hatsopoulos et al., 1998; Nirenberg and Latham, 1998; Panzeri et al., 1999a, 2010; Golledge et al., 2003; Schneidman et al., 2003; Ince et al., 2010). The former is simply computed as described above on the original data, which are made of a combination of synergy coefficients simultaneously acquired in each trial and thus contain trial-to-trial correlation between synergy activation coefficients. The information in absence of correlations can be denoted as *I*_ind_ (*T*;*T*^*P*^), the “ind” subscript indicating that it is built from data that are made to be distributed independently at fixed task. *I*_ind_ (*T*;*T*^*P*^) can be computed again with the procedure described above, but applying it to combinations of synergy coefficients obtained after “shuffling” the data at fixed task, i.e., combining synergy values into non-simultaneous arrays each taken (randomly and without replacement) from different trials in which task *t*was performed (Ince et al., 2010; Panzeri et al., 2010). This shuffling preserves the marginal distributions of the activation of each synergy, and only changes the distribution of their joint observations. All subsequent decoding and information analysis were performed on the shuffled data, and then information results were averaged over the outcome of 50 independent random shufflings. Note that shuffling is done both on the test and training data for the decoder, thus in this way the effect of correlations is removed from both the decision boundaries determined by the decoder (using the training data) and the actual data to be decoded (test data). In the following, for brevity we will denote *I*_ind_ (*T*;*T*^*P*^) simply as *I*_ind_.

**Figure 2:**
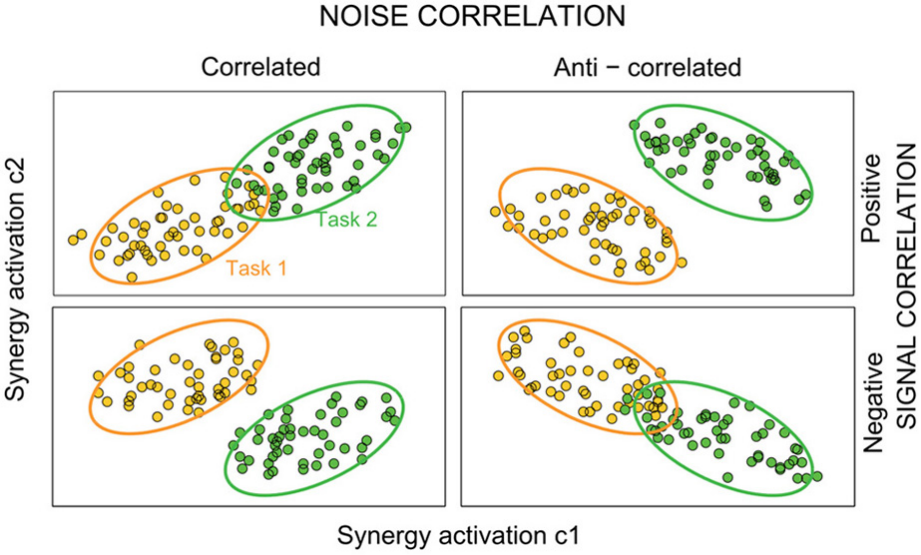
**The effect of interplay of signal and noise correlations between synergies on task information.** Each panelsketches joint distributions of activations of two hypothetical synergiesduring two different tasks (data for task one and two are plotted in orange and green color, respectively). The dots represent a hypothetical scatterplot from single-trial activations to the given task, and each ellipse denotes 95% confidence limits. In the upper panel, there is positive signal correlation (i.e., individual synergy activations to each task are positive correlated), whereas in the lower panels there is negative signal correlation. Positive noise correlations correspond to ellipses aligned along the diagonal. The more the ellipses are elongated, the stronger the noise correlation. The sign of noise correlations between the joint responses differs across columns of this figure (noise correlation is positive in the left column and negative in the right column). In this figure, noise correlations are task independent—equally strong across stimuli (all the ellipses within a panel have the same elongation). In general, if noise and signal correlation have opposite signs, the effect of correlations increases the information about tasks, because the joint response probabilities to each task become more separated. If instead noise and signal correlation have the same sign, tasks are less discriminable.

From the theoretical point of view, correlations between synergy coefficients can either increase (i.e., *I* > I_ind_) or decrease (i.e., *I* < I_ind_) information with respect to the case in which their marginal distributions are the same but there is no correlation (Pola et al., 2003). If noise correlations increase the information carried by the muscle synergies, one may speculate that correlations between synergy activations may be useful in describing the salient task-to-task variations of muscle activation patterns. Whether or not correlations increase or decrease the information depends on several factors (Oram et al., 1998; Panzeri et al., 1999a; Averbeck et al., 2006). The first is the stimulus modulation of the strength of noise correlation: strongly task modulated correlation tend to increase the information, because their task-to-task modulation tends to further pull apart task-conditional distributions of joint synergy activations (Panzeri et al., 1999a; Pola et al., 2003). The second is the interplay between the sign of signal and noise correlations. If signal and noise correlation have opposite signs, noise correlations increase task discriminability compared to what it would be if noise correlations were zero because in such case they tend to pull apart task-specific joint distributions (Oram et al., 1998; Abbott and Dayan, 1999). If, instead, noise and signal correlations have the same sign, then noise correlations decrease information, and task are less discriminable than the zero noise correlation case. For intuitive illustration of these effects, see Figure 2.

### Generation of simulated EMG data

To investigate whether the synergy extraction algorithm used (NMF) affects the correlational structure of the identified synergies, we tested it on EMG data generated from muscle synergies with known task-dependent correlations. To this end, we simulated EMG data from synchronous synergies using the model introduced in Delis et al. (2013). Briefly, the data simulated the activation of 10 muscles used for executing 50 repetitions of each one of 2 motor tasks (T1 and T2). To execute each motor task, the first synergy was activated by a scalar coefficient drawn from a uniform distribution in the [0,1] interval. The activation coefficients of the second synergy were correlated with the ones of the first synergy with a correlation coefficients *r*_1_ for task T1 and *r*_2_ for task T2 respectively. We varied the levels and signs of *r*_1_ and *r*_2_ as shown in Table 1 to generate four different datasets and ran the synergy extraction 50 times for each dataset.

**Table 1:** **Correlation coefficients of the original (left columns) and the reconstructed (right columns) activations of the two simulated synergies for tasks T1 (*r*_1_) and T2 (*r*_2_)**.

**Original**		**Reconstructed**	
***r***_1_	***r***_2_	***r***_1_	***r***_2_
0.77	0.64	0.62 ± 0.06	0.42 ± 0.08
−0.82	−0.43	−0.78 ± 0.01	−0.42 ± 0.02
0.70	−0.74	0.68 ± 0.01	−0.73 ± 0.01
0.79	0.10	0.69 ± 0.05	−0.08 ± 0.08

### Experimental procedures

To test the methodology presented above and gain more insights about its potential usefulness, we applied it to physiological EMG data recorded in an experiment that has been presented before (Delis et al., 2013). In brief, the experimental dataset that will be used throughout the Results Section was composed of the EMG activity recorded from nine upper body and arm muscles during execution of arm pointing movements in the horizontal plane (see for a detailed description). Four participants were asked to perform center-out (forward, denoted by fwd) and out-center (backward, denoted by bwd) one-shot point-to-point movements between a central location (P0) and four peripheral locations (P1-P2-P3-P4) evenly spaced along a circle. In total, the experimental protocol specified 4 targets × 2 directions = 8 distinct motor tasks denoted by T1, T2, …, T8. Each task was composed of 40 trials. Such a relatively high number of repetitions of each task was useful for evaluating the impact of trial-to-trial variability on the combination of muscle activation patterns.

Body kinematics was recorded by means of a Vicon (Oxford, UK) motion capture system with a sampling rate of 100 Hz. The kinematics data were low-pass filtered (Butterworth filter, cut-off frequency of 20 Hz) and numerically differentiated to compute tangential velocity and acceleration. For each movement, we measured movement onset time, movement end time, maximum speed, maximum acceleration and their times of occurrence. Movement onset and movement end were identified as the times in which the velocity profile of the fingertip superseded 5% of its maximum. Subjects performed all motor tasks at a variety of speeds ranging from normal to very fast. For example, movement durations for subject AK ranged from 182 ms to 651 ms. Across subjects, the mean movement duration varied from 370 ms to 560 ms.

To identify muscle synergies from these data, we used the time course of EMG activity of all recorded muscles in all individual trials for each task.

## Results

In this section, we illustrate the application of our method to muscle synergies identified from the EMG data recorded during the performance of point-to-point reaching movements in different directions in the horizontal plane. We first present extensive results of our analysis applied to the synchronous synergies of all subjects and then refer briefly to its application to the time-varying synergies.

### Impact of the synergy extraction algorithm on correlations

**Figure 3:**
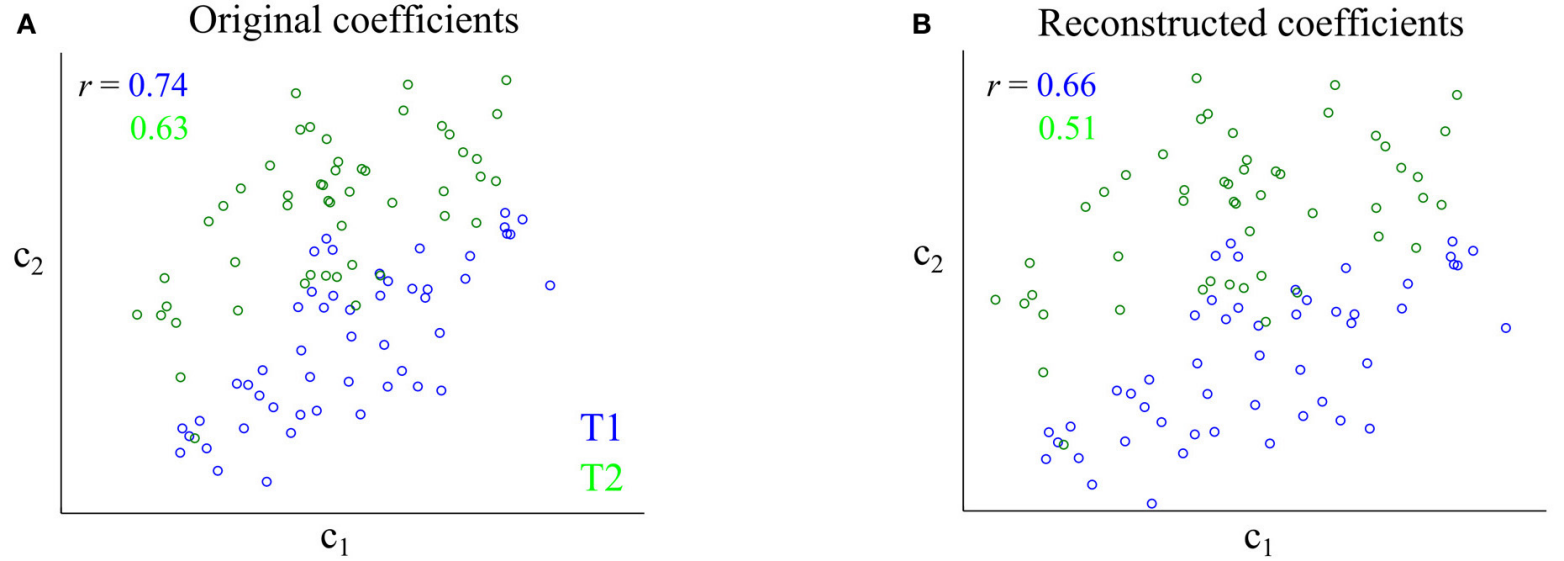
**Impact of synergy extraction using NMF on the correlations between synergy activations. (A)** Scatterplot of the original activations of the two simulated synergies for two tasks (T1 and T2). **(B)** Scatterplot of the activations of the two synergies as identified by the NMF algorithm. The *r* values report correlation coefficients at fixed task. Noise correlations are slightly weaker after application of NMF to the data.

Before applying our methodology to experimental data, we tested the ability of the NMF algorithm to identify correctly the correlational structure in the synergy space. Thus, we simulated EMG data from synergies with known correlations (see Materials and Methods) and checked whether the output of the NMF algorithm represented reliably the correlations that were present in the underlying synergies. An illustration of the impact of synergy extraction on the correlations between synergy activations is shown in Figure 3. In this case, the original activations of the two synergies are highly positively correlated for both tasks considered (Figure 3A). The NMF algorithm identified correctly the two synergies as well as the positive noise correlations between their activation coefficients (Figure 3B). However, the strength of noise correlations was slightly underestimated by NMF.

We then investigated what happened when simulating data with different levels of either positive or negative correlations between the synergy activations. The results are summarized in Table 1. In all cases, the NMF algorithm recovered the original synergies and the sign and strength of the signal and noise correlations between the activation coefficients. The signal correlations were reconstructed accurately in all cases (*p* > 0.05, *t*-test), whereas the reconstructed noise correlations were in general slightly but significantly (*p* < 0.05, *t*-test) lower compared to the original activations (see Table 1). In sum, the application of NMF to our simulated data estimated correctly signal correlation and underestimated slightly noise correlation among the activation coefficients of the extracted synergies.

### Muscle synergy identification and task modulation

We extracted synchronous muscle synergies from the recorded and pre-processed EMG data and used our information-based methodology to identify the smallest synergy sets that explain, for each subject, all task-discriminating information in the synergy space. In brief, the method first computes the information about task carried by each synergy; then computes the information from a set of synergies starting with the most informative ones and then including other synergies until they stop providing additional information. Since this selection is done using simultaneous (non-shuffled data), this set of synergies contains all information about task that can be extracted from the EMG data, including the information that is transmitted by correlations among synergy activations. Considering synergies belonging to this set (rather than for example including other synergies that explain some variance in the data but do not add any additional task discriminability) ensures that we can work on a compact set that yet contains all relevant variables for task discrimination.

Results of this synergy selection using this information criterion were very similar to the synergy selection that we obtained in a recent study (Delis et al., 2013) on the dataset using a slightly different and slightly less powerful criterion (we considered only percentage of correct decoding—rather than Shannon information—as the task discriminability metric in the above procedure). We will therefore present the selected synergy sets briefly and we refer to Delis et al. (2013) for more details. We found (Figure 1) that our method selected four synergies for two subjects (AK, ES) and three for the other two (AM, AB)- Figure 1C. These synergies not only carried all information about differences across tasks but also explained a high percentage of the variance of the recorded EMG data (see VAF curves in Figure 1C). Most of the extracted synergies were highly similar across subjects and each one had a distinct functional role in movement execution (Figure 1A). S1 activated mainly muscles flexing the shoulder, S2 consisted mainly of muscles extending the elbow, S3 had high activations for elbow flexors and S4 activated highly the shoulder extensors. The results were relatively robust across subjects: three of the four synergies (S1, S2, S3) were identified in all subjects and the fourth (S2) was present in two of them (AK, ES). In the other two subjects, the elbow extensors were activated by other synergies. By examining the trial-averaged synergy activations (Figure 1B), we observe that all synergies were shared across the eight motor tasks but their activation levels differed, which led to a high task-discrimination power in the muscle synergy space. Task modulations of individual synergies were robust across subjects, indicating a consistent mapping between synergy activations and task identification.

### The interplay of signal and noise correlations and its effect on task-discriminating information

The previous section considered the tuning of individual synergy coefficients to task. We next wanted to investigate the nature of the joint (rather than the marginal) distributions of synergy coefficients across trials. In other words, we asked if the activation of any given synergy depended only in the task or also on the particular level of activations of other synergies in the same trial. We also asked whether single-trial correlations between synergy activations may affect task discriminability.

**Figure 4:**
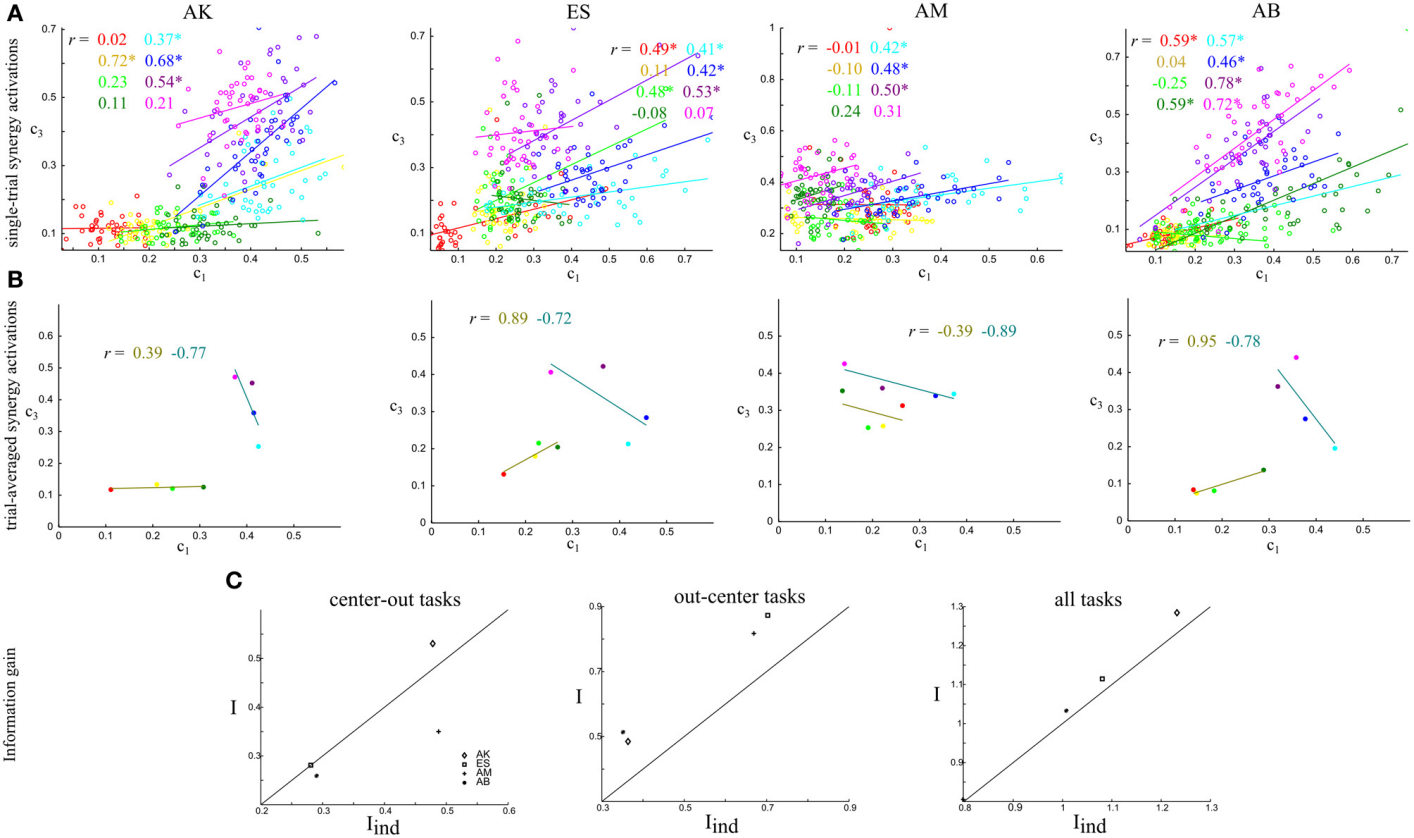
**Quantifying noise and signal correlations between the activations of synergies S1–S3, and assessing their impact on information about the task. (A)** Scatterplots of the integral of the single-trial activations of synergies S1–S3 across the eight tasks performed. Each one of the four panels corresponds to the data of one subject. Different colors indicate different tasks and the straight lines are the best-fit lines for the distribution of points for each task. The *r* values report correlation coefficients and the ^*^ denotes statistical significance at *p* < 0.05. **(B)** Scatterplots of the task-averaged activations of synergies S1–S3. The two straight lines are the best-fit lines for the distribution of points for the center-out and out-center tasks respectively. Conventions are the same as in **(A)**. **(C)** Comparison of the information carried by the activations of synergies S1–S3 when taking into account correlations (*I*) and when ignoring them (*I*_ind_). The scatterplots show I vs. I_ind_ across subjects. We plot the 45°-slope line for comparison purposes. Left: Comparison of information about the center-out tasks. Middle: Comparison of information about the out-center tasks. Right: Comparison of information about all tasks.

To gain more insights into trial-to-trial variations of synergy activations, we first illustrated scatter plots of the integrals of the single-trial activation coefficients for one pair of synchronous synergies (S1–S3). In Figure 4A, different tasks are color-coded to show the distribution of synergy activations at fixed task. Colored straight lines indicate the principal axis of dependence and the reported values correspond to the correlation coefficient for each task. Positive noise correlations are reflected in elliptic distributions aligned along the diagonal. The more the ellipses are elongated, the stronger the noise correlation.

The strength of noise correlations between synergy activations varied significantly depending on the task. For example, activations of the S1–S3 synergy pair were strongly positively correlated for the out-center tasks (T5-T6-T7-T8), but they were almost uncorrelated for the center-out tasks T1-T2-T3-T5 (Figure 4A). This finding was robust across all four subjects. Thus, these two synergies were differentially coupled across tasks, suggesting that they constitute a functional pair whose single-trial interactions are relevant for the performance of a subset of motor tasks. Theoretical studies demonstrate that the modulation of correlations across different experimental correlates can only increase the information carried about the external correlates, with respect to the case of no correlation or non-modulated correlations (Panzeri et al., 1999a; Pola et al., 2003). These theoretical considerations suggest that the observed task modulation of noise correlations of synergy pairs increase the task discriminability.

To evaluate further the impact that noise correlations may have on the information carried by this pair of synergies, we also examined their signal correlation. As discussed in Materials and Methods, signal correlations of sign opposite to that of noise correlations are best suited to increase task information (Figure 2). We considered separately signal correlations between out-center and center-out tasks, as they seemed to give a different pattern of correlations. The joint distribution of the trial-averaged synergy activations across out-center tasks of synergiesS1 and S3 shows that signal correlation was negative robustly across subjects (Figure 4B). In other words, out-center tasks that elicited on average a higher activation of synergy S1 for a subgroup of tasks tended to elicit a lower average activation for synergy S3 (Figure 4B). Taking into account that noise correlations for center-out tasks were consistently positive, we expected a significant increase of information about center-out tasks due to correlation. We verified this by comparing the amount of information about center-out tasks that can be obtained by observing S1 and S3 simultaneously to the information *I*_ind_ obtained from data shuffled by pairing S1 and S3 activations from randomly selected trials to the same task. We remind that, while *I* contains all information carried by synergy coefficients, including the effect of their correlations, *I*_ind_ is computed from data manipulated to preserve the same marginal distributions of each synergy but without any correlation. Indeed, we found (Figure 4C middle) that the information carried by S1 and S3 about out-center tasks was, for each subject, significantly higher (22.5 ± 1.5%, paired *t*-test, *p* < 0.01) than the information *I*_ind_ that neglected the effect of correlations. This suggests that correlations among such synergies enhance the amount of discriminability between out-center tasks.

We then considered signal and noise correlations of S1 and S3 activations among center-out tasks. For the center-out tasks the sign and strength of signal correlations varied across subjects being mostly positive and in general lower in magnitude than the signal correlation among out-center tasks. Hence, we expected the effect of correlations between S1 and S3 on information about center-out tasks to be lower than the effect on out-center tasks. Indeed, by computing the information carried by these two synergies when correlations are taken into account and comparing it with that of the “independent” model (i.e., when we remove noise correlations by shuffling), we found that correlations did not contribute much to the discrimination of the center-out tasks (Figure 4C left). This means that noise correlations between S1 and S3 have a positive effect on the identification of the out-center tasks, but essentially no effect for the center-out ones. Over all tasks, there was a significant increase of 4 ± 0.5% (paired *t*-test, *p* < 0.05) in the information about all tasks carried by the activation of S1 and S3 due to correlation between S1 and S3 (Figure 4C right).

**Figure 5:**
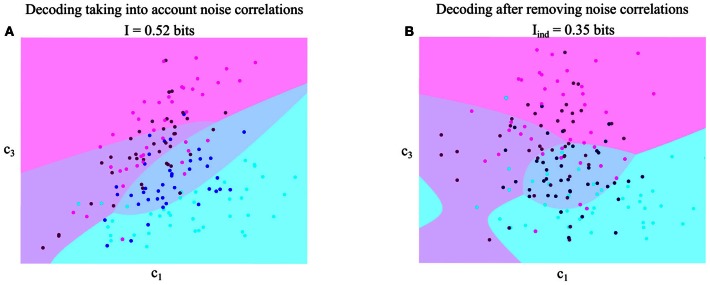
**Illustration of the effect upon decision boundaries and joint synergies activation distributions of the removal of noise correlations in task discrimination.** Decoding the four out-center motor tasks (T5-T6-T7-T8) using synergies' S1–S3 activation coefficients taking into account correlations **(A)** or after removing them **(B)**. For a given trial to be decoded, the activation coefficients of the synergies are represented as a point in the 2-dimensional space. The color of each point indicates the actual task which this trial corresponds to. The quadratic discriminant algorithm has divided the space into four regions, one for each motor task. The trial is assigned to the task indicated by the color of the region on which the point lies.

To gain more insights into the impact of correlations on task discrimination, we illustrated graphically the decoding procedure for these two synergies and the four out-center tasks using data from one subject (AB). Figure 5A shows the integral of activations of S1 and S3 when correlations were present (Figure 5A) and when they were removed by shuffling (Figure 5B). In Figure 5, the QDA determined the decision boundaries for classifying the trials to the motor task performed, and as a result, separated the 2-dimensional spaces into 4 regions, one for each task. Each point is assigned to the task represented by the colored region on which it lies. The presence of noise correlations resulted in elliptic distributions of the synergy activations at fixed task, which were identified well by the QDA algorithm (Figure 5A). Thus, 57% of the trials were correctly decoded and 0.52 bits of information about the task were carried by the co-activation of these two synergies. On the contrary, after eliminating correlations, the distributions of data points were more circular which led to a higher overlap between different tasks and, as a result, to decreased decoding performance (52%) and information (0.35 bits), as shown in Figure 5B.

**Figure 6:**
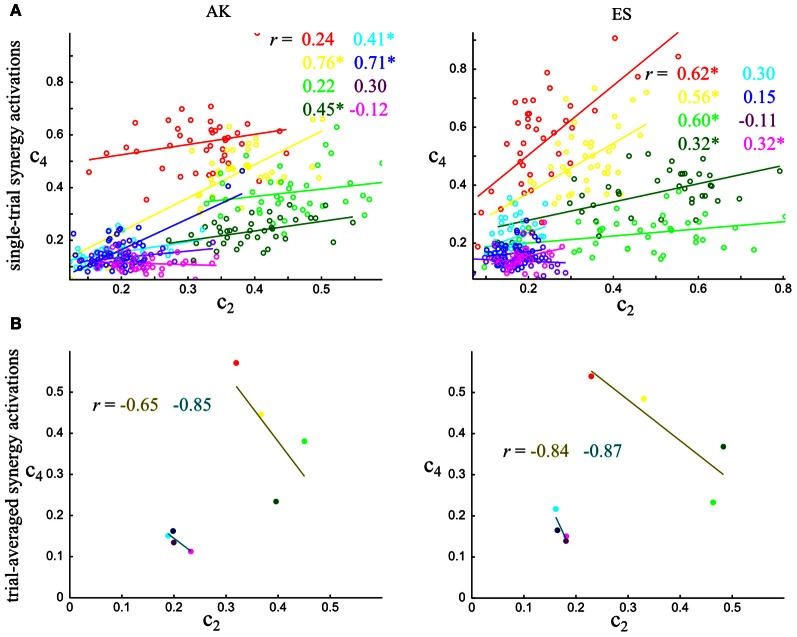
**Quantifying noise and signal correlations between the activations of synergies S2–S4, and assessing their impact on information about the task. (A)** Scatterplots of the integral of the single-trial activations of synergies S2–S4 across the eight tasks performed. **(B)** Scatterplots of the task-averaged activations of synergies S2–S4. The *r* values report correlation coefficients and the ^*^ denotes statistical significance at *p* < 0.05. Conventions are the same as in Figure 4.

Following this, we investigated how these patterns of signal and noise correlations generalized to other synergy pairs. We found (Figure 6) that the synergy pair S2–S4 exhibited a pattern of positive noise and negative signal correlations for the center-out tasks (but not for the out-center tasks). This finding was true for both subjects (AK, ES) for whom we detected four synergies describing all the task related information. We quantified the information gain also in this case and found that information about the task increased by 17% on average when correlations were kept in the analysis.

For all other synergy pairs, noise correlations were mostly weak (*r* < 0.35) and non-significant. The few cases of strong noise correlations were not robustly found across subjects and/or tasks. Regarding signal correlations, activations of synergies S3–S4 were robustly negatively correlated across all eight tasks considered (*r* = − 0.80 on average) (as can also be observed by their task-tuning curves in Figure 2) but did not show strong noise correlations at fixed task (*r* = 0.14 on average).

**Figure 7:**
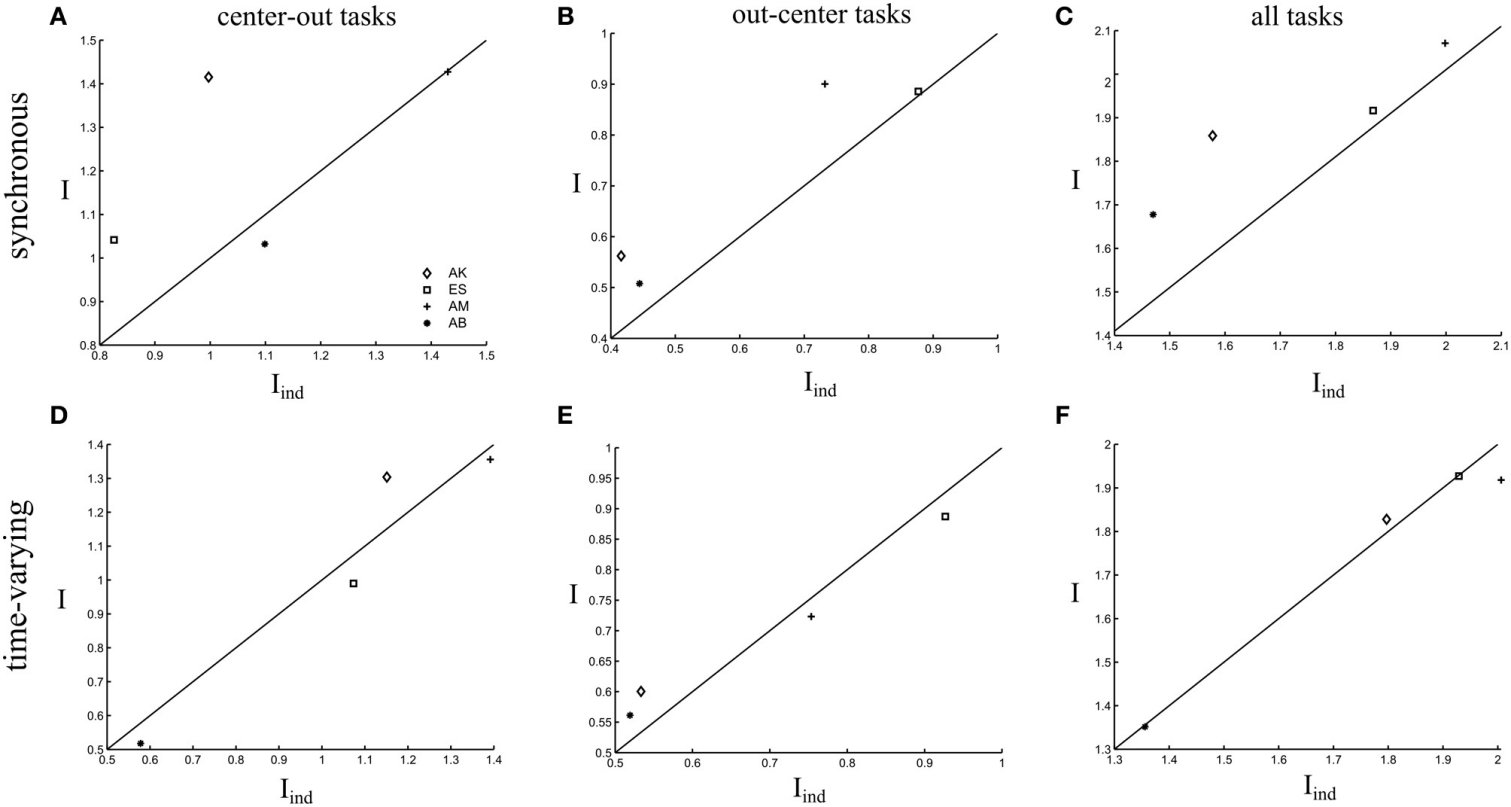
**Comparison of the total information carried by the activations of all synergies when taking into account correlations (*I*) and when ignoring them (*I*_ind_).** The scatterplots show the information values in the *I*-I_ind_ space for all subjects. We plot the 45°-slope line for comparison purposes. **(A,D)** Comparison of information about the center-out tasks. **(B,E)** Comparison of information about the out-center tasks. **(C,F)** Comparison of information about all tasks.

Finally, we evaluated quantitatively the contribution of correlations between all synergies explaining task-to-task differences for the four recorded datasets. When considering only the center-out tasks, noise correlations had a slightly detrimental effect on task-discriminating information coding for the two subjects that used three synergies to perform the tasks but contributed positively for the other two subjects (Figure 7A). Across subjects, the average information increase when considering correlations was 11 ± 5.5% but the increase was not significant at the population level (paired *t*-test, *p* > 0.1). For out-center tasks, there was a significant information gain of 11 ± 2.5% (paired *t*-test, *p* < 0.1) when including correlations across all four subjects (Figure 7B). We should also note that task discrimination of out-center tasks was poorer than for the center-out for all datasets. Thus, the presence of noise correlations could play a role in improving task discriminability in cases where confusions between tasks are more likely. Overall, the presence of noise correlations resulted in a significant increase of 9 ± 1.5% (paired *t*-test, *p* < 0.05) in the total task-discriminating information that was present in the muscle synergy decompositions (Figure 7C).

These findings suggest that single-trial correlations between muscle synergy activations increase task-related differences in muscle activation patterns.

### Dependence of synergy co-activations on movement speed

**Figure 8:**
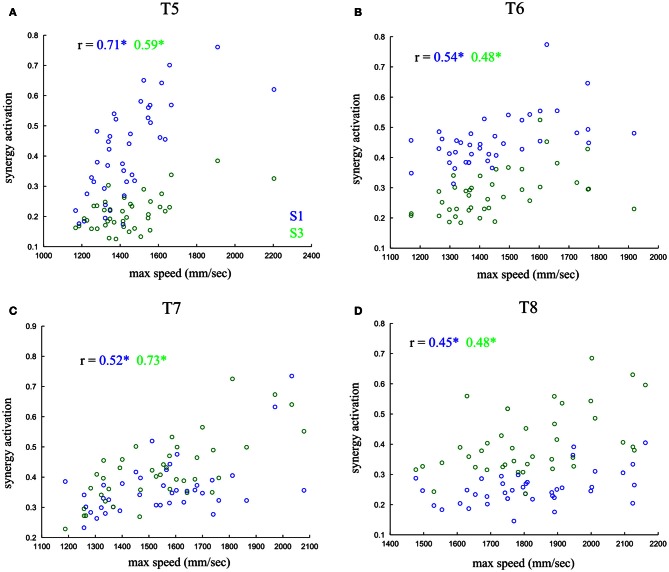
**Dependence of synergy activations on movement speed.** Scatterplots of the single-trial activations of synergy S1 (blue) and S3 (green) with respect to the max movement speed in each trial at fixed task. The four panels **(A–D)** correspond to the four out-center tasks. The r values report correlation coefficients and the ^*^ denotes statistical significance at *p* < 0.05.

We next asked whether noise correlations among synergy activations may in part reflect the effect of the trial-to-trial covariations of a movement parameter. To answer this, we analyzed the relationship of synergy activations with single-trial kinematic parameters of the movements, such as movement duration, maximum speed and acceleration. Interestingly, our correlation analysis revealed a significant dependence of the activations of synergies S1–S3 on the speed with which the movement was performed for all out-center tasks. Figure 8 reports scatterplots of the activations of each one of the two synergies at fixed task with respect to maximum movement speed for one subject. These results generalize across all subjects: significant activation-speed correlations were found in 10 out of 16 cases (4 center-out tasks times 4 subjects) for synergy S1 and 11 out of 16 for synergy S3 (*p* < 0.05). Accordingly, activations of synergies S2–S4 were correlated with movement speed for the four out-center tasks for both subjects that used S2 (5/8 and 6/8with significant correlations, *p* < 0.05). Therefore, the positive synergy correlations at fixed task explain a big part of the variability in the speed with which the task was executed.

### Extension of results to time-varying synergies

Although we illustrated our methodology and discussed our findings only within the synchronous synergy framework so far, the method's applicability can be readily extended to the time-varying synergies. Here we illustrate this extension by decomposing the same dataset in time-varying rather than synchronous synergies.

**Figure 9:**
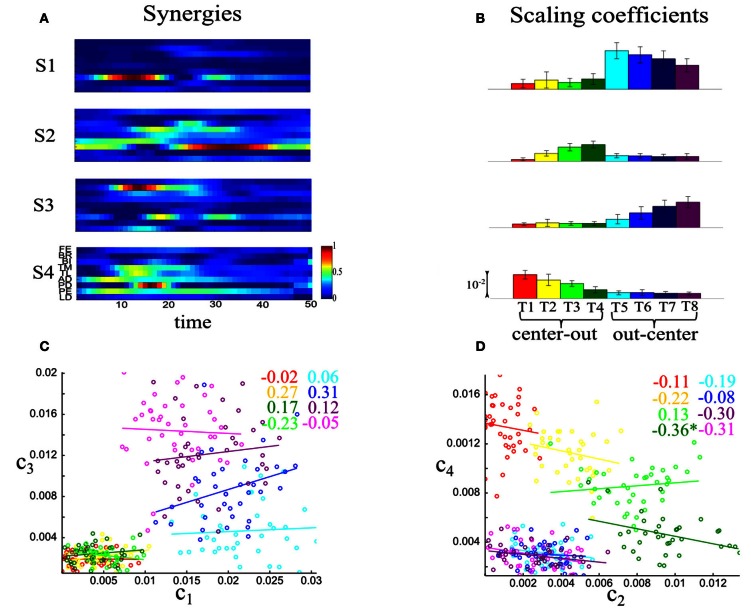
**Quantifying noise correlations between time-varying synergy activations. (A)** The four time-varying synergies obtained from the experimental data of one subject (AK) as four matrices each representing the activity of nine shoulder and elbow muscles over 50 time steps. **(B)** Histograms of the coefficients activating the four synergies across the eight motor tasks performed. Histograms are plotted as means ± SDs across all trials to the given task. **(C)** Scatterplot of the activation coefficients of synergies S1–S3 across the eight tasks performed. **(D)** Scatterplot of the activation coefficients of synergies S2–S4 across the eight tasks performed. The reported values are correlation coefficients and the ^*^ denotes statistical significance at *p* < 0.05. Overall, noise correlations are much weaker than for the synchronous synergy activations.

We found that our synergy selection method identified the same number of time-varying synergies as the number of synchronous synergies for all the four datasets tested. Figure 9A shows the four time-varying synergies identified from one subject's dataset. Similar task modulations of average synergy activations were present also for this model (Figure 9B). Also in this case, all synergies participated in the execution of the eight motor tasks varying their activation levels in a task-dependent manner. However, when examining the impact of trial-to-trial correlations between synergies, we found weak noise correlations among the scaling coefficients activating the time-varying synergies. An illustrative example of the strength of noise correlations between time-varying synergy activations is shown in Figures 9C,D. To compare with the synchronous synergy case presented above, we depict scatterplots for the synergy pairs S1–S3 and S2–S4. In this case, noise correlations are non-significant for almost all tasks. Therefore, these correlations had no impact on task discrimination (they did not increase task-discriminating information, Figure 7F). In particular, correlations increased information about the task only in one of the four datasets for both center-out and out-center tasks, whereas they reduced or did not affect information for the other three datasets (see Figures 7D–F). This finding indicates that correlated activations of time-varying synergies contribute less to task identification with respect to the modulations of individual synergies.

## Discussion

In this article, we extended a previously-developed single-trial task decoding formalism (Delis et al., 2013) to derive a novel methodology that evaluates quantitatively the impact of trial-to-trial correlations among muscle synergy co-activations on the task-to-task differences in patterns of muscle activation. Our aim was to suggest and test methodological ideas to answer an important question concerning task information coding in the CNS, and more precisely in the context of neuromuscular synergies: Do correlations between synergy activations play a role indiscriminating motor tasks?

### Methodological developments

The methodological approach that we suggested is derived from the literature in neural population coding, in which the problem of the role of trial-to-trial correlations among groups of simultaneously active neurons have been hotly investigated (Averbeck and Lee, 2006; Averbeck et al., 2006; Ecker et al., 2011). The approaches have been useful both to clarify the conditions in which correlation may increase or decrease information (Oram et al., 1998; Panzeri et al., 1999a; Schneidman et al., 2003; Latham and Nirenberg, 2005; Averbeck et al., 2006; Oizumi et al., 2010) and to indicate the potentially large impact of correlation on the accuracy of population codes especially when considering large number of cells (Zohary et al., 1994; Salinas and Sejnowski, 2001; Averbeck et al., 2006; Schneidman et al., 2006; Pillow et al., 2008; Ince et al., 2010; Oizumi et al., 2011). This has led several authors to propose that the ability of cortical circuits to modify, regulate or tune their correlations is an important feature of cortical functional organization (Salinas and Sejnowski, 2001; Ecker et al., 2010; Kumar et al., 2010; Renart et al., 2010).

In this article we discussed how to adapt this approach to EMG recordings. From the methodological point of view, we needed to make some progress before being able to adapt previous neural approaches to EMG recordings. First, we needed a method to select correctly the number of synergies to be used, which is one of the free parameters of the analysis. Here, we took the approach of selecting this number as to give the smallest possible set of variables that carries all task-discriminating information. Second, the sampling issues in computing information are more severe in EMG experiments than in most experiments used to study neural coding in peripheral system or anaesthetized animals, and synergy activations are analog variables rather than yes/no variables (like for spiking neurons). Here we addressed these problems by using an intermediate “decoding” step to project the relatively high-dimensional space of all synergy activations into the task space, and we computed the effect of correlations using data manipulations to destroy them rather than computing their effect with analytical techniques (Panzeri et al., 1999a; Pola et al., 2003). The approach was overall robust and allowed to study, with realistic datasets, the effect of correlation on the task information on decompositions of arrays of up to nine muscles. We feel that the coupling of NMF procedures with information algorithms presented here could be a valuable tool not only for evaluation of muscle synergies, but also for the analysis of large scale simultaneous recordings of neural activity, as it combines two powerful data compression procedures to describe compactly the information content of large datasets. As such, it could be fruitful to further the progress of our understanding of the functional role of correlations among groups of neurons. Here, we used this approach to quantify, using EMGs recorded in a simple reaching task, the amount of task-discriminating information carried by trial-to-trial correlations among synergy activations. We found that, overall, the presence of correlations between the integrated activation of synchronous synergies enhanced information significantly. Correlations among activations of synchronous synergies increased information about all tasks by 9%, and increased information about out-center reaching tasks by approximately 15%. While they may seem relatively small amounts, we must take into account that the effect of correlation might have been negative, and that this was a simple task that involved only a small set of synergies. Previously documented scaling properties of the impact of correlation of information with the number of elements in the array suggests that more complex tasks involving larger number of synergies may have larger increases of information due to correlation. All in all, these preliminary results suggest that the study of impact of correlations among synergies may be helpful in future experiments to better understand how neural drives need to interact with each other to optimize motor strategies.

### Robustness of synergy activation correlations to factorization errors

The applicability of the proposed methodology to evaluate the role of correlations in synergy activations relies on the ability of the synergy extraction algorithms (NMF here) to correctly estimate the correlational structure of the underlying muscle synergy activations. We evaluated this issue using simulated data with known correlations between synergy activations and found that the output of the NMF algorithm recovers correctly the sign and strength of signal correlations and underestimates only slightly the strength of noise correlations. It is interesting to consider the effect of these small errors in correlation estimation introduced by the NMF on estimating the impact of correlation activation in task discriminating information. In the EMG dataset that we analyzed, the increase in task-discriminating information because of correlation was due to the combination of negative signal plus positive noise correlation. Hence, the effect of the algorithm's artifact (slight reduction in noise correlations only) would go in the direction of slightly reducing the effect of correlation on task-discriminating information with respect to the true value. Thus, the conclusion that correlations between synergy activation coefficients increase task discriminating information seems a genuine property of the data that cannot be attributed to artifacts in correlation estimation due to the NMF decomposition.

### Potential origins of noise correlations

It is tempting to speculate that noise correlations may emerge as a result of the mechanisms implemented by the CNS to guarantee reliable movement reproducibility. For example, correlations among synergies coordinate their relative activation levels in order to stabilize limb movement against the detrimental effect of motor noise which leads to trial-by-trial variability in the neural motor commands. A possible way to achieve this is by using positive noise correlations to regulate the level of muscle co-contraction during movement execution. Put simply, by increasing activation of two muscle groups simultaneously, many muscles are highly activated leading to an increase of the stiffness of the moving limb which enhances movement stability. An example of such case is given in Figure 4A. Synergies S1 and S3 are comprised of shoulder flexors and elbow flexors respectively. Their positive noise correlations during performance of tasks T5-T6-T7-T8 (combined with the negative signal similarity) result in the co-contraction of these muscles which enhances task discrimination. As these two groups of muscles act on different joints, their positive noise correlations may suggest a cross-joint coupling of synergy activations to achieve specific task goals. In other words, such anatomical muscle groups may be coupled together to form new functional muscle synergies for some subsets of tasks. Alternatively, because of the vicinity of these muscle groups, we could think that these positive correlations may result from crosstalk artifacts. However, presence of crosstalk would imply strongly positive correlations for all tasks performed, which is not the case here. Instead, the different levels of muscle co-contraction may serve as a “tag” for the target reached in each trial. These differences might be explained by the inertial properties of the arm, for which some movement directions require less muscle effort than others (Gordon et al., 1994).

We also showed that a large part of the synergy co-variations at fixed task explained the variability in the speed with which different trials were performed. Since the task (pointing to a target) did not impose movement speed, the variability in the muscle synergy activations that captures trial-to trial speed variations can be considered as irrelevant for the set of tasks considered (Scholz and Schoner, 1999). Hence, a large part of the variability in the muscle synergy space may correspond to these redundant (task-irrelevant) dimensions in task space (Todorov and Jordan, 2002; Todorov et al., 2005). This interpretation relates closely to the “uncontrolled manifold” (UCM) concept (Scholz and Schoner, 1999) and its application to muscle synergies (Krishnamoorthy et al., 2003). In broad agreement with the UCM hypothesis, our results show that the noise correlation between synergy activations accounts in part for the trial-to-trial variability of a task-irrelevant parameter, as is movement speed in our experiment. As such, our methodology may contribute to answer questions related to the identification of hypothesized task performance variable. Whereas applying the UCM concept to EMG data is not straightforward because partitioning the synergy space variance into components that affect and do not affect the task variable is tricky [in Krishnamoorthy et al. (2003), the method relies on the computation of the Jacobean of the mapping from synergy activations to task variable and its null-space], our method provides a principled and robust information-theoretic approach to address the problem. Extending our method to actually handle the case of task-execution variables such as the reach endpoint coordinates is a future and promising line of research.

Other neurophysiological evidence suggests that noise correlations between synergy activations may arise during execution of correcting movements. Corrections in reaching movements driven by feedback mechanisms have been shown to be described by the superposition of the muscle synergies that are used for unperturbed reaching (D'Avella et al., 2011). Although trial-to-trial variability was not considered in this study, synergy superposition may reflect robust muscle synergy correlations in single trials that are identified by the synergy extraction algorithm as invariant patterns. Thus, the open-loop muscle synergies may be coupled to form new muscle patterns that are appropriate for the accomplishment of the corrected motor task. Further support to this consideration comes from a study showing that sensory feedback can couple two independently-organized synergies (or uncouple two centrally-coupled synergies) by modulating activation of each synergy independently (Cheung et al., 2005). These findings suggest that synergy activations may be coordinated in single trials by central mechanisms.

### Correlations in synchronous vs. time-varying synergy activations

Our results indicate a more important role of noise correlations in the synchronous synergy model compared to the time-varying synergies. Intuitively, this can be explained if we take into account the functionality of each type of synergies. On the one hand, synchronous synergies consist in functional groups of muscles whose activities co-vary across all tasks (Tresch et al., 2006). In our data, each one of the four synchronous synergies includes muscles that have the same functional role (either flexion or extension of the limb) and act on the same joint. As such, these groups have to be recruited simultaneously in many flexible combinations to perform a variety of motor tasks. Furthermore, recruitment of each synergy in every single trial depends crucially on the recruitment of other synergies, as muscle groups counteract or complement the activation of other muscle groups (e.g., an agonist-antagonist pair of synergies). This explains the existence of large trial-by-trial interactions between synchronous synergy activations and points out the importance of their function. On the other hand, time-varying synergies consist in spatiotemporal patterns of muscle activities that are invariant across tasks (D'Avella and Tresch, 2002; D'Avella and Bizzi, 2005). Although this formulation constitutes a very compact representation of muscle activities in single trials (2 single-trial parameters per synergy), it does not allow much flexibility in reusing muscle synergies across tasks because of the merging of spatial and temporal properties in one unique pattern. This results in the identification of more “task-specific” synergies that participate in the execution of only a subset of tasks. More importantly, activation of only one time-varying synergy can be sufficient for the execution of a task without rendering necessary the simultaneous and interactive activation of other synergies. This is because every time-varying synergy specifies an entire muscle activity waveform for each muscle. As a result, in this framework, task differences are mainly described by the activation of different time-varying synergies and thus, task-discriminating information is carried mainly by the modulations of individual time-varying synergies. Nevertheless, these conclusions are drawn on a somewhat simple dataset, and further investigations should be performed on more complex data involving more motor tasks (also more muscles, more targets, more speeds etc.). The present study mainly aimed at establishing a systematic and principled methodology to address such questions.

### Conflict of interest statement

The authors declare that the research was conducted in the absence of any commercial or financial relationships that could be construed as a potential conflict of interest.
